# Progressively Prolonged PR Interval and Aortic Abscess

**DOI:** 10.7759/cureus.45341

**Published:** 2023-09-16

**Authors:** Murtaza Ali, Gohar Rundhawa, Rahul Kashyap, Michael N Vranian

**Affiliations:** 1 Department of Internal Medicine, Wellspan York Hospital, York, USA; 2 Department of Research, Wellspan York Hospital, York , USA; 3 Department of Cardiology, Wellspan York Hospital, York, USA

**Keywords:** mssa, conduction abnormalities, pr interval prolongation, infective endocarditis, aortic abscess

## Abstract

Regular electrocardiogram (ECG) monitoring in patients with endocarditis of the aortic region is a simple yet effective approach to help evaluate for the development of aortic abscess. It is important to recognize this condition as it carries a high morbidity and mortality. We report a case of a 62-year-old Caucasian female diagnosed with methicillin-sensitive *Staphylococcus aureus *(MSSA) bacteremia with mitral and aortic endocarditis. Progressive PR prolongation prompted re-evaluation, ultimately finding the progression of a new aortic abscess, changing the patient’s care pathway. With a standardized approach of obtaining regular ECGs in patients with aortic endocarditis, it is possible to identify the progression of aortic valve endocarditis, thereby lowering the risk of morbidity and mortality.

## Introduction

Infective endocarditis is the inflammation and infection of the endocardium most often involving valvular structures. Predisposing risk factors include congenital heart defects, valve replacements, intravenous drug use, intra-cardiac devices, central line placements, immunosuppression, and recent dental or surgical procedures. The incidence is roughly 15 people per 100,000 people [1]. Paravalvular extension, including the development of an abscess, fistula, and heart block occurs in 10% to 20% of patients with native valve endocarditis, most commonly due to aortic and mitral valve involvement [2]. It is associated with significant morbidity and mortality and must be treated with prompt surgical evaluation. The proximity of the atrioventricular (AV) node and the aortic root leads to various forms of heart block as one of the earliest manifestations of abscess formation in that area. PR interval monitoring can provide insight into the development of this devastating complication [3].

## Case presentation

A 62-year-old female with a past medical history of hypertension, end-stage renal disease on hemodialysis through an internal jugular tunneled dialysis catheter, and ovarian cancer presented with weakness and lethargy. She was having persistent fevers to 102°F, no leukocytosis but a C-reactive protein (CRP) of 386 mg/L. She was found to have methicillin-sensitive *Staphylococcus aureus *(MSSA) bacteremia from a dialysis catheter infection.

She had acute embolic infarcts in her spleen and brain. She subsequently had intracerebral hemorrhage due to hemorrhagic conversion of her cerebral infarcts. A large mobile superior vena cava vegetation was noted on imaging likely due to her hemodialysis line that was subsequently replaced. In the setting of persistent MSSA bacteremia and septic emboli, she underwent transthoracic and transesophageal echocardiographic (TTE, TEE) evaluation demonstrating mitral and aortic valve endocarditis with competent valves and without abscess formation or perforation. She was treated with IV antibiotics on presentation. The patient continued to remain persistently bacteremic and febrile to 102°F despite antibiotics prompting surgical evaluation.

**Figure 1:**
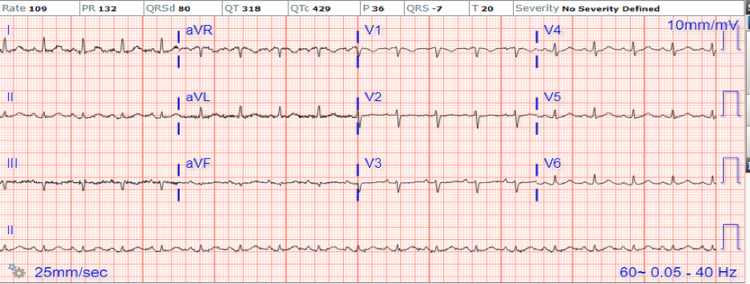
Initial ECG on admission: Sinus tachycardia, PR interval 132 ms

**Figure 2:**
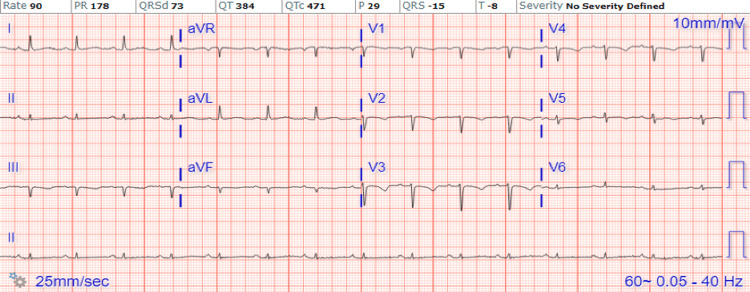
ECG 13 days later: Normal sinus rhythm, PR interval 178 ms

Given the hemorrhagic conversion of the cerebral infarcts, surgery was felt too high risk, and initial conservative management followed by planned surgery a few weeks later was felt the safest course of action. Despite the improvement of fever, the decline in CRP to 128 mg/L, and normal white cell count, serial ECGs obtained during the hospitalization showed PR interval prolongation from 132 ms to 178 ms (Figures 1, 2). The patient had no electrolyte abnormalities or acidosis that could explain the PR prolongation. 

**Figure 3:**
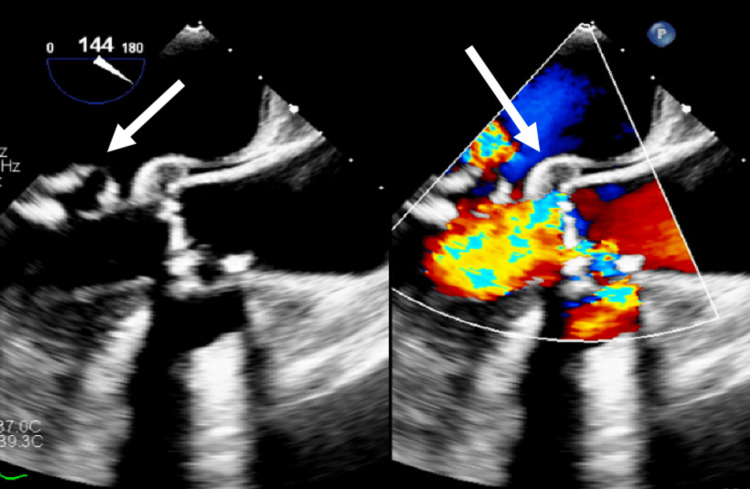
Mitral valve vegetations and aortic abscess Both leaflets of the mitral valve are involved with endocarditis with highly mobile vegetations. The most notable vegetation is seen on the posterior leaflet which is 1.7 x 1 cm. The anterior leaflet vegetation is smaller - roughly 0.75 x 0.5 cm. The right coronary cusp has a definite vegetation. The left coronary cusp has a definite vegetation.

**Figure 4:**
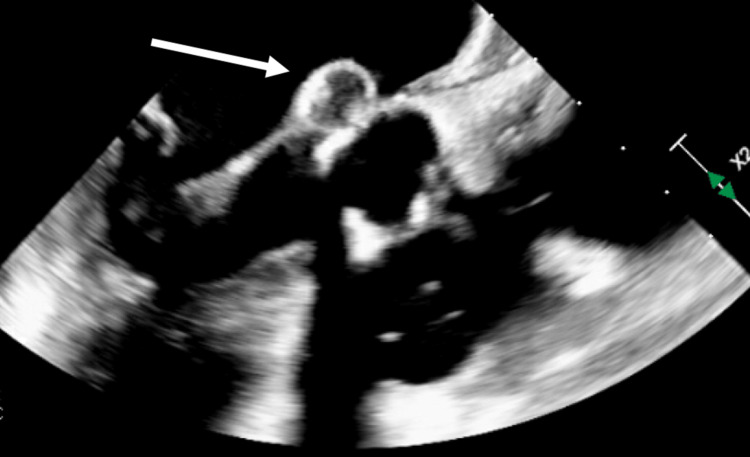
Aortic abscess Aortic abscess is adjacent to the vegetation on the anterior leaflet involving the aortomitral curtain.

This prolongation of PR interval prompted a repeat TEE, 11 days after the initial TEE. This showed the development of a new aortic abscess demonstrating the failure of antibiotics and the need for urgent surgery (Figures 3, 4). The patient subsequently underwent replacements of the aortic valve and mitral valve as well as repairs of the aortic annulus and superior vena cava. Ultimately, she was found to have MSSA and treated with naficillin for 6 weeks, followed by amoxicillin and repeat blood cultures to ensure bacterial clearance.

## Discussion

Infective endocarditis of aortic and prosthetic valves is most commonly associated with perivalvar infections, such as abscesses and fistulas [3]. Periannular abscess is a dangerous complication of infective endocarditis, occurring in up to 46% of cases involving the aortic valve [4]. Anatomically, the aortic valve and AV node are located close to the intraventricular septum, which contains the conduction system. It is this relationship that explains why pathology involving the aortic valve can lead to complete heart block or conduction abnormalities [5]. During a heart block with a prolonged PR interval, a pressure gradient between the left ventricle and left atrium develops causing the early partial closure of the mitral valve in diastole. Subsequently, atrial contraction after nonconducted P wave leaves the mitral valve open causing diastolic regurgitation as a consequence [6]. Occurrence and persistence of conduction disturbances are not sensitive indicators of perivalvar infection but have a specificity of 85%-90%. The utility of a cost-effective and easily performed ECG holds significant value as daily ECG monitoring has good specificity for predicting aortic abscess formation and should prompt more urgent echocardiographic evaluation. It is recommended that TEE must be considered in all endocarditis patients with previously unrecognized conduction disturbances, aortic or prosthetic valve involvement, or indications for valve replacement [7]. An approach that involves frequent ECGs will allow for the detection of prolongation of the PR interval as well as the recognition of conduction abnormalities. It can clue in a physician to repeat echocardiograms and evaluate for complications. This will allow for appropriate treatment and involvement of the surgical team.

## Conclusions

The ability to identify endocarditis complications through regular ECGs with subsequent echocardiogram evaluation is underused in clinical practice and serves as an incredible tool to help identify complications such as aortic abscess. Monitoring the ECG is recommended for PR interval prolongation or the development of arrhythmias, which are indicators of endocarditis complications. If implemented in regular practice with prompt surgical intervention, the morbidity and mortality of such conditions would likely be improved.
